# Purified diets containing high levels of soluble fiber and grain-based diets promote similar gastrointestinal morphometry yet distinct microbial communities

**DOI:** 10.1128/aem.01552-24

**Published:** 2024-10-24

**Authors:** Elaine M. Glenny, Jintong Liu, Harlyn G. Skinner, Tori L. McFarlane, Kylie K. Reed, Alyssa Weninger, Zorka Djukic, Michael A. Pellizzon, Ian M. Carroll

**Affiliations:** 1 Department of Nutrition, University of North Carolina at Chapel Hill, Chapel Hill, North Carolina, USA; 2 Department of Nutrition, Center for Health Promotion and Disease Prevention, University of North Carolina at Chapel Hill, Chapel Hill, North Carolina, USA; 3 Department of Molecular Genetics and Microbiology, Duke University, Durham, North Carolina, USA; 4 Center for Gastrointestinal Biology and Disease, Univeristy of North Carolina at Chapel Hill, Chapel Hill, North Carolina, USA; 5 Research Diets, Inc., New Brunswick, New Jersey, USA; The Pennsylvania State University, University Park, Pennsylvania, USA

**Keywords:** intestinal microbiota, fiber, soluble fiber, insoluble fiber, purified diets, grain-based diets

## Abstract

Dietary fibers play a crucial role in shaping the gut microbiome and influencing gastrointestinal (GI) physiology. Grain-based diets (GBDs) are widely used in rodent studies, but their utility is limited due to batch-to-batch variability resulting from inconsistent ingredients. Purified diets (PDs) are composed of only known and refined ingredients and offer a solution to the constraints of GBDs. This study aimed to identify a combination of dietary fibers in a purified diet (PD) that promotes optimal murine gut morphometry and a diverse intestinal microbial community. Male C57BL/6J mice were fed either two grain-based diets (GBDs) or four PDs with varying fiber compositions for 28 days. Mice consuming PDs lacking soluble fiber had more gonadal fat (*P* < 0.05), shorter small intestines (*P* < 0.05), and lighter ceca (*P* < 0.05) compared with those fed the LabDiet 5001 GBD. Increasing the proportion of soluble fibers in PDs progressively reduced microbial diversity in the cecum and colon. Multidimensional scaling analysis revealed distinct microbial communities in the cecum and colon between mice fed GBDs and PDs (*P* < 0.05). Differential abundance analysis identified relatively more *Family XII UCG 001* and less *Lactococcus* in mice fed GBDs relative to mice consuming PDs (*P* < 0.05). While no PD recapitulated the gut microbial composition of GBDs, PDs with high soluble fiber content best preserved GI morphometry. These findings underscore the importance of considering diet as an experimental variable and highlight the need for a PD formulation that combines the benefits of GBDs on GI health and microbial richness.

**IMPORTANCE:**

Dietary fibers are essential for maintaining gut health. Insoluble fibers aid in fecal bulking and water retention while soluble fiber is a fermentative substrate for intestinal microbial communities. Grain-based diets (GBDs) are commonly used in preclinical research but the variability in ingredients across batches impedes reproducibility. Purified diets (PDs), which are composed of highly refined ingredients, pose a potential solution but the most widely used low-fat control PDs contain no soluble fiber. This study intended to identify a PD with a combination of fibers that promotes murine gut health and microbial diversity. A PD with optimal fiber composition would aid in the standardization and reproducibility of studies investigating intestinal physiology and the gut microbiota.

## INTRODUCTION

Intestinal microbial communities shape gastrointestinal (GI) physiology in health and disease (1–3). Multiple host and environmental factors including age, geographic location, sex, antibiotic use, and diet influence intestinal microbial composition and consequent community functions (4). Acute diet switches and chronic dietary patterns particularly impact the gut microbiota, as even short-term dietary changes modify gut microbial communities in humans (5, 6). Additionally, preclinical research demonstrates analogous findings—dietary factors such as fat content can alter gut microbiota composition in mice (7).

Fiber is a principal dietary constituent that significantly influences intestinal microbial community structure and is critical for promoting GI health (8). Insoluble fiber is a fecal bulking and water retention agent that alters GI transit time (9). Conversely, gut microbes that encode the required enzymes can ferment soluble fibers to short-chain fatty acids to provide an energy source for intestinal microbial communities and the colonic epithelium (10). A deficit in dietary soluble fiber may necessitate enteric microbes to extract energy from the colonic mucus barrier, thereby increasing the host's susceptibility to pathogenic infection (11, 12). Thus, fiber is a crucial nutrient for both intestinal microbial communities and the host.

Diets used in preclinical experiments are typically either grain-based diets (GBDs) or purified diets (PDs). GBDs, informally known as chow, are generally manufactured from agricultural and animal by-products such as ground corn, ground oats, alfalfa meal, soybean meal, and ground wheat (13). While economical, GBD ingredient composition—including dietary fibers—will vary across batches (13). In contrast to GBDs, PDs are defined diets with highly refined ingredients and therefore contain precise amounts of each ingredient and nutrient to negate variability between batches and increase reproducibility across animal studies (14, 15). However, the most popular PDs, including the AIN-76A and AIN-93 series (AIN-93G and AIN-93M), contain only 5% total fiber as cellulose, which is an insoluble fiber and mostly non-fermentable (16–18). Chassaing et al. demonstrated that PDs containing only cellulose increased adipose stores and promoted abnormal GI morphometry in mice relative to mice fed a GBD and that these observations could be reversed with the replacement (or addition) of inulin to the PDs (19)—others have noted similar findings (20–22).

The goal of the current study was to identify a combination of dietary fibers in a PD that promotes optimal murine gut morphometry and an ecologically diverse intestinal microbial community. We evaluated the benefits of different fiber mixtures by: (i) characterizing the impact of six different murine diets (two distinct GBDs and four PDs with varying fiber composition) on murine adiposity and GI morphometry; (ii) comparing the microbial composition of mice consuming these six diets across three distinct GI niches (cecum, colon, and feces); and (iii) identifying specific bacterial genera that were differentially abundant among mice fed the different diets. As balanced diets for humans contain a mix of soluble and insoluble fibers, we hypothesized that diets with a greater diversity of soluble and insoluble fibers would support the most diverse intestinal microbial community and promote normal GI morphometry in mice.

## RESULTS

### Amount and variety of dietary fiber impacts food consumption, GI morphometry, and adiposity

**Fig 1:**
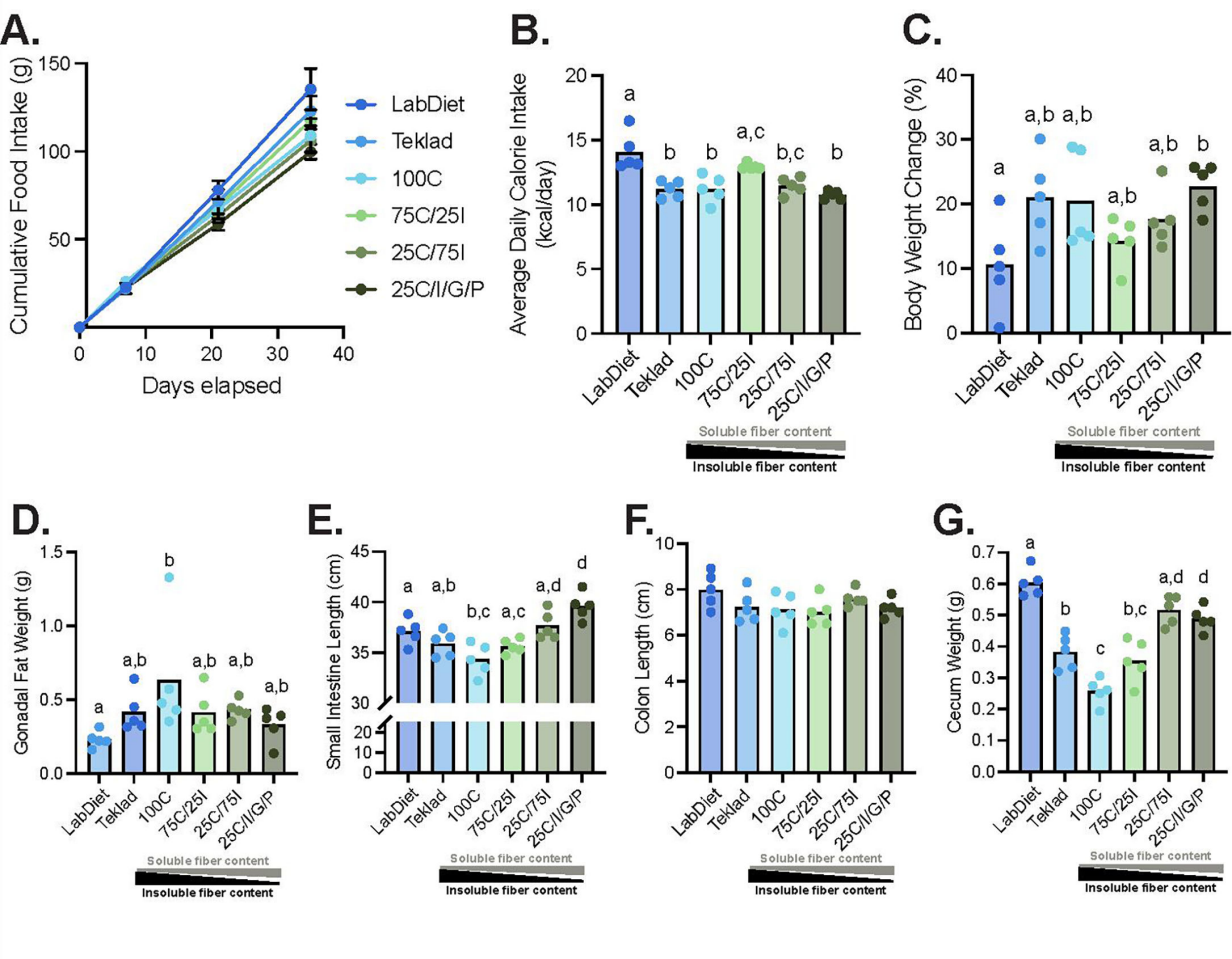
The type and amount of dietary fiber impact food consumption, intestinal morphometry, and adiposity. Cumulative food intake (error bars indicate SD) (**A**), average daily calorie intake (**B**), percent body weight change (**C**), gonadal fat weight (**D**), small intestine length (**E**), colon length (**F**), and cecum weight (**G**) are shown for mice in each dietary group. Statistical differences were determined using a one-way analysis of variance (ANOVA) with Tukey's post-hoc test. Each experimental group was compared to all other groups with different letters denoting a significant difference (*P* < 0.05). Specifically, there is a statistical difference between groups if a group does not share a letter with another group.

Cumulative food intake varied across diet groups (Fig. 1A). Mice consuming LabDiet 5001 had a higher average daily calorie intake than mice assigned to Teklad 2020X and every other PD except 75C/25I (Fig. 1B). Mice that consumed a greater quantity and mix of soluble fibers (25C/I/G/P) ate fewer calories relative to mice fed a greater amount of insoluble fiber (75C/25I) (Fig. 1B).

Mice assigned to LabDiet 5001 gained less weight compared with mice fed the 25C/I/G/P diet (Fig. 1C), although no significant differences in absolute body weights were detected between diet groups at the end of the experiment. Additionally, mice assigned to the 100C diet had more gonadal fat than mice fed LabDiet 5001 diet (Fig. 1D). No other differences were observed in body weight or adipose stores across the other diet groups.

Relative to mice fed LabDiet 5001, small intestines were longer in mice fed the 25C/I/G/P diet and shorter in those consuming the 100C diet (Fig. 1E). Increasing the amount and variety of dietary soluble fiber impacted small intestinal length as mice fed the 25C/I/G/P diet exhibited longer small intestines than mice consuming the 75C/25I and 100C diets (Fig. 1E). No differences were observed in colon length (Fig. 1F). Ceca from mice fed LabDiet 5001 were heavier than mice fed Teklad 2020X (Fig. 1G). In the PDs, increasing the amount of soluble fiber in the diet was associated with heavier ceca (Fig. 1G).

### Increasing amounts of soluble dietary fiber reduce gut microbial diversity in a stepwise manner

**Fig 2:**
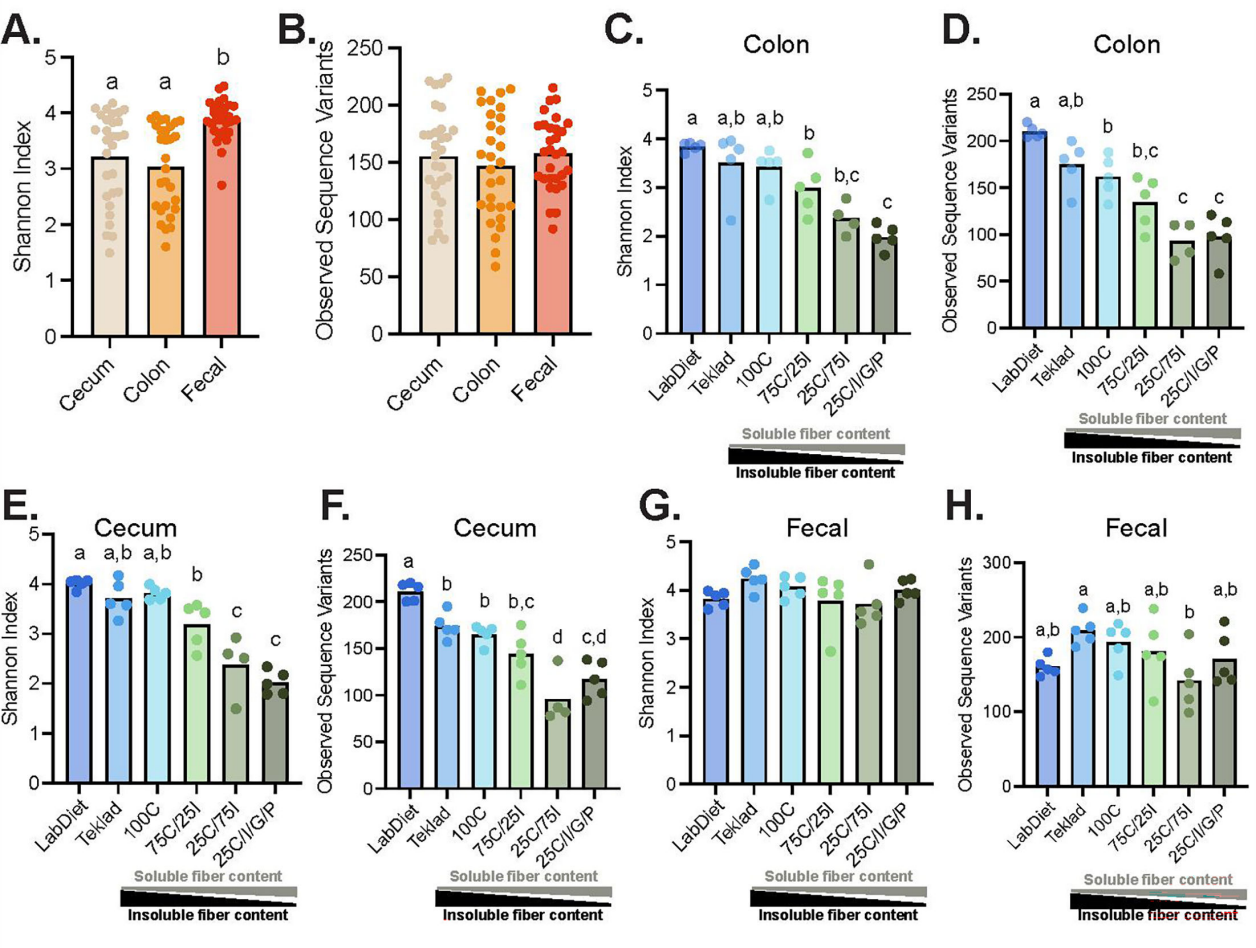
Increasing amounts of soluble dietary fiber reduce gut microbial diversity. Shannon index (**A**) and number of SV (**B**) across three GI niches. Shannon index (**C**) and number of SV (**D**) in colon samples among diet groups. Shannon index (**E**) and number of SV (**F**) of cecal samples among diet groups. Shannon index (**G**) and number of SV (**H**) of fecal samples among diet groups. Statistical differences were determined using a one-way ANOVA with Tukey's post-hoc test. Each experimental group was compared to all other groups with different letters denoting a significant difference (*P* < 0.05). Specifically, there is a statistical difference between groups if a group does not share a letter with another group.

Microbial communities were characterized across three GI niches at the end of the study (day 35)—cecum, colon, and feces. Microbial richness (determined by Shannon index and observed number of sequence variants, SV) did not differ between cecal and colonic samples; however, the fecal microbiota was more diverse based on Shannon index (Fig. 2A and B). Contrary to our hypothesis, increasing the amount of soluble fiber was associated with a reduction, rather than an increase, in α-diversity. In the cecum and colon, the greatest microbial diversity (based on Shannon index) was observed in mice fed either a GBD or the 100C diet lacking soluble fiber while mice ingesting the most soluble fiber (25C/75I and 25C/I/G/P) had the lowest cecal α-diversity (Fig. 2C through E). Additionally, mice fed Teklad 2020X exhibited lower observed SVs in their cecal contents compared with those fed LabDiet 5001 (Fig. 2F). In fecal samples, while there were no differences for Shannon diversity across any diet, mice fed the Teklad GBD had a greater number of observed SVs relative to mice consuming the 25C/75I PD (Fig. 2G and H).

### Fiber composition shapes gut microbial communities

**Fig 3:**
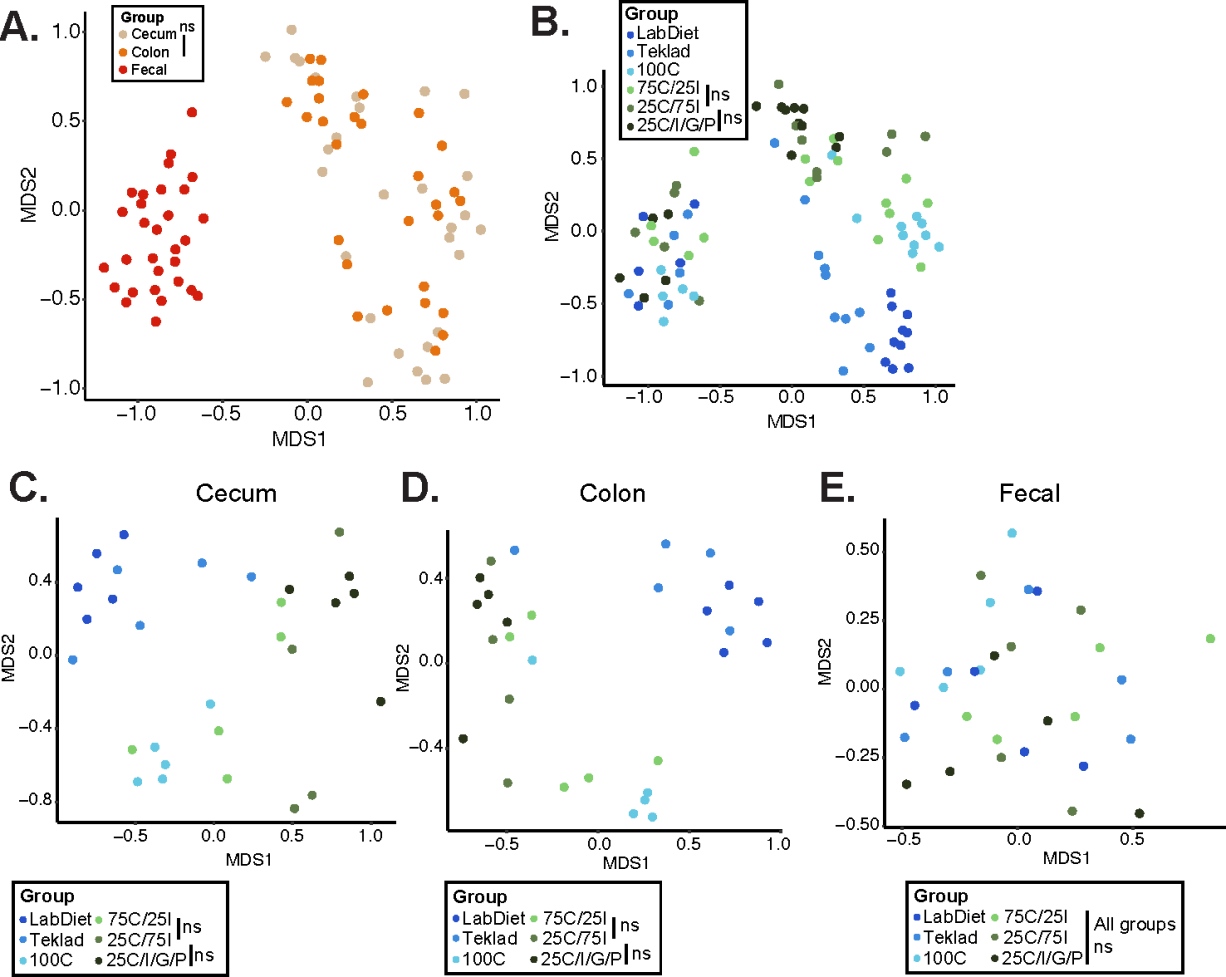
GI niche and dietary fiber influence microbial community composition. MDS plot using Bray-Curtis dissimilarity distances of microbial communities from the cecum, colon, and feces colored by diet group (**A**). MDS plot using Bray-Curtis dissimilarity distances of all GI niches combined (**B**), only cecal (**C**), only colonic (**D**), and only fecal (**E**) microbial communities colored by diet group. *P* < 0.05 between all diet groups (PERMANOVA) unless indicated as non-significant (ns).

Fecal microbial communities were not different prior to mice being assigned a diet on day 7 (Fig. S1A and B). Multidimensional scaling (MDS) plots using Bray-Curtis dissimilarity distances revealed significantly different microbial community structures in the cecum and colon compared with feces (Fig. 3A). MDS plots combining samples across all three GI niches revealed that microbial communities were distinct between mice fed GBDs and PDs and this observation was more pronounced within the cecum and colon (Fig. 3B through E). Mice consuming the highest amounts of soluble fiber (25C/75I and 25C/I/G/P) had different cecal and colonic microbial communities relative to mice fed PDs with less soluble fiber (Fig. 3C and D). Adjusting the amount of soluble and insoluble fiber (75C/25I vs. 25C/75I) did not result in distinct microbial communities while reducing insoluble fiber and adding a more diverse profile of soluble fibers (25C/IG/P vs. 75C/25I) influenced microbial composition (Fig. 3C and D). Interestingly, fecal microbial communities did not segregate based on diet group (Fig. 3E). Overall, these data illustrate that diet type (i.e., PD vs. GBD) along with changes to soluble fiber contents (particularly increasing the number of soluble fibers) exerted a strong influence on shaping the microbial communities in the cecal and colonic niches.

### GBDs differentially influence the composition of the gut microbiota relative to PDs

**Fig 4:**
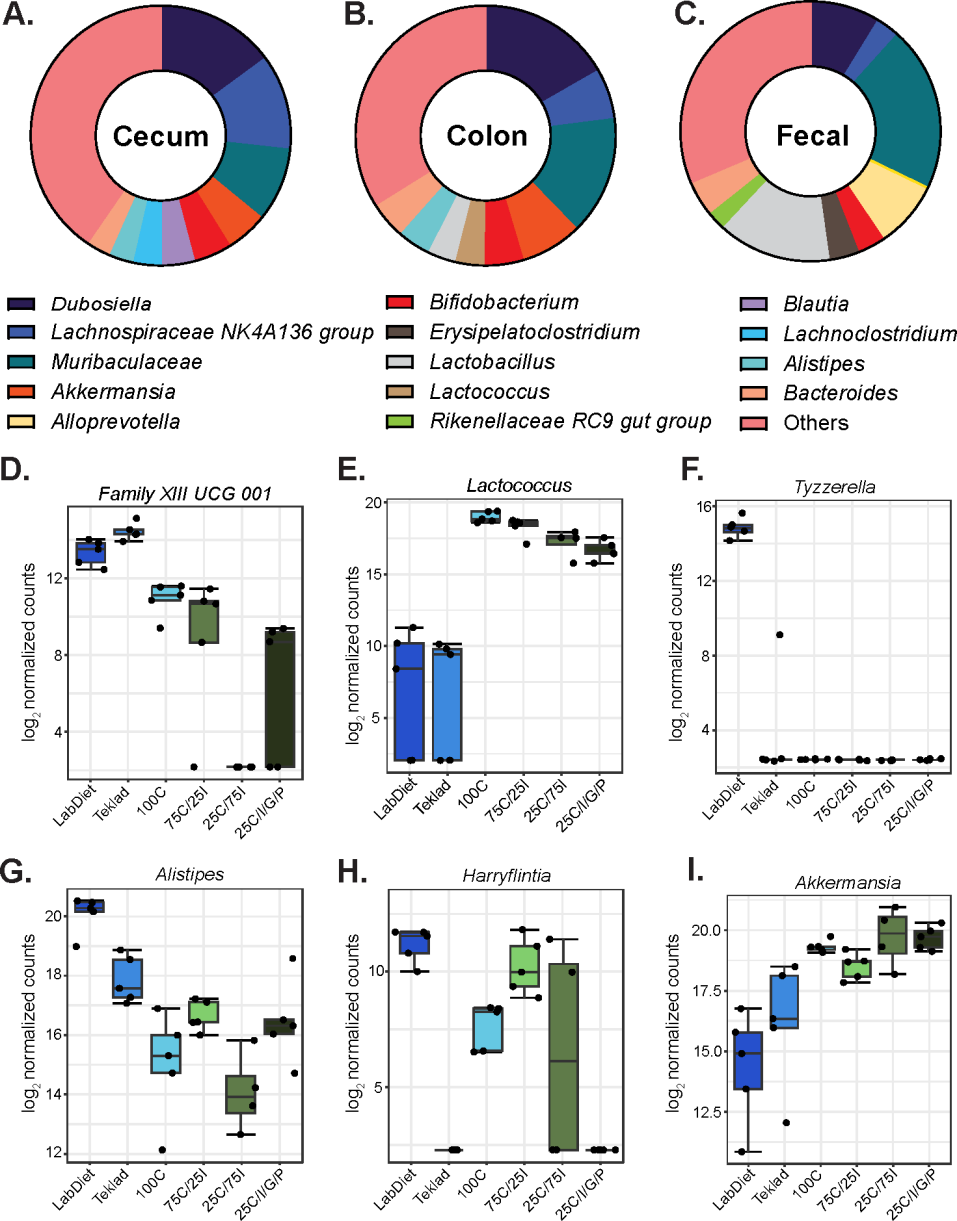
Diet types enrich for specific bacterial genera. Relative abundance of the top nine most abundant bacterial genera in cecal (**A**), colonic (**B**), and fecal (**C**) microbial communities. Relative abundance in the cecum among diet groups for *Family XIII UCG 001* (**D**), *Lactococcus* (**E**), *Tyzzerella* (**F**), *Alistipes* (**G**), *Harryflintia* (**H**), and *Akkermansia* (**I**). D-I whiskers extend to the smallest and largest value that are no further than 1.5*IQR (inter-quartile range) from the first and third quartiles, respectively.

As cecal and colonic microbiotas exhibited a more similar composition than fecal microbial communities and as the cecum has the greatest microbial metabolic activity, we performed a differential abundance analysis on cecal samples to identify specific bacterial genera driving differences in community composition among diet groups. Although the relative abundance of bacterial taxa varied between GI niches (Fig. 4A through C), the amount and type of fiber influenced the relative abundance of specific microbial genera (Tables S1 and S2). Based on a LEfSe differential abundance analysis, *Family XIII UCG 001*, *Lactococcus*, *Tyzzerella*, *Alistipes*, *Harryflintia*, and *Akkermansia* were the top six genera most likely to explain differences between groups based on the false discovery rate (FDR) q value (Fig. 4D through I; Tables S1 and S2). Specifically, the relative abundance of *Family XIII UCG 001* was higher in the cecum of mice consuming GBDs than PDs while the abundance of the *Lactococcus* genus was higher in the cecum of mice consuming PDs than GBDs (Fig. 4D and E). The highest levels of *Tyzzerella* were found in the ceca of mice fed LabDiet 5001 (Fig. 4F). *Alistipes* was more abundant in mice fed LabDiet 5001 relative to all other diets while *Akkermansia* was more abundant in mice fed PDs compared with mice consuming LabDiet 5001 (Fig. 4G and I).

## DISCUSSION

Consistent with Chassaing et al., we found that mice consuming a PD lacking soluble fiber (100C) accumulated more adiposity relative to mice fed LabDiet 5001—a GBD (19). Additionally, despite consuming more calories, mice on LabDiet 5001 gained the least weight over the 28-day experimental period. Moreover, mice consuming LabDiet 5001 exhibited longer small intestines than mice fed the 25C/I/G/P diet and shorter than those fed the 100C diet. Not only did our current study reproduce major findings from Chassaing et al. but we also expanded upon this study by evaluating a second GBD (Teklad 2020X) to delineate whether the previous observations were specific to LabDiet 5001 or generalizable to the broader category of GBDs. Unexpectedly, unlike mice on LabDiet 5001, mice that consumed Teklad 2020X did not have less gonadal fat than mice fed any of the four PDs. We also found that although gut microbial communities clustered according to GI niche, cecal and colon microbiotas from mice consuming GBDs clustered separately from mice consuming PDs—an observation not recapitulated in the fecal microbiotas.

These results suggest that diet is an experimental variable that should be carefully considered in preclinical studies. While both GBDs and PDs significantly, and uniquely, impacted weight gain, adiposity accumulation, GI morphometry, and intestinal microbial communities, open-source PDs offer the opportunity to reduce experimental variability and increase reproducibility across studies by using defined ingredients. Indeed, many rodent studies employ PDs to promote metabolic disease such as obesity, nonalcoholic steatohepatitis, and cardiovascular disease yet use GBDs for the control group (15, 23, 24). However, differences in the dietary formulations between PD and GBD—known and unknown—confound data interpretation in these studies. A recent study demonstrated that the lack of soluble fiber in PDs, rather than the addition of fat, drove alterations in the gut microbiota of mice consuming a diet commonly used to study obesity (24). Another study reported that the microbial communities between mice fed GBDs and PDs were more distinct than those between mice fed a high-fat PD and a low-fat PD (25). Our findings demonstrate that diet type (GBD or PD) significantly influences microbial community composition, with GBDs and PDs showing greater similarity within each diet type than to the other diet type. This underscores the importance of controlling for diet in these studies.

We found that the addition of soluble fibers in PDs reduced microbial α-diversity in cecal and colonic microbial communities. This observation agrees with reports demonstrating a progressive reduction in α-diversity as the contribution of soluble fiber to a diet increases (21, 22). Reduced microbial diversity is typically associated with a low-fiber, high-sugar diet and linked to metabolic syndrome and inflammatory bowel diseases in preclinical and clinical studies (26). Additionally, a meta-analysis of nutritional interventions modulating dietary fibers across 64 human trials reported no significant association between fiber and microbial α-diversity (27). It was therefore not surprising that the addition of soluble fibers to rodent diets had a beneficial impact on the GI tract (based on gross morphometry) yet reduced microbial richness. One hypothesis may be that insoluble fibers, to a greater extent than soluble fibers, influence microbial richness as the PDs used in this study contained increasing quantities of insoluble fiber as the soluble fiber quantity decreased. In one report, the addition of soluble fiber sources (i.e. inulin, psyllium, or combination of both) to PDs containing cellulose had no effect on cecal microbial α-diversity in male mice following 10 weeks on the diet (28). Future studies are needed to test this hypothesis and to uncover a potential mechanism.

Our study demonstrated that GI niche (cecum, colon, and feces) drove differences in microbiota composition. Additionally, gut microbiotas segregated within the cecum and colon based on whether mice were consuming GBDs or PDs. Altering the amount of soluble fiber did not have a significant impact on gut microbial community composition. Specifically, mice consuming PDs with variable amounts of soluble fiber (75C/25I, 25C/75I, and 25C/I/G/P) had distinct gut microbial communities relative to mice fed GBDs or a PD with only insoluble fiber (100C). This phenomenon was observed in cecal and colonic microbial communities, but not in the feces. The lack of diet-driven differences in gut microbiota composition within fecal samples questions the utility of this niche when investigating diet-microbiota interactions; however, the benefit of using fecal samples to investigate longitudinal changes in microbiotas may outweigh this limitation. One limitation of our study is that mucosal-associated microbial communities were not investigated. Given the proximity of mucosal-associated microbial communities to the host gut epithelial barrier (29, 30), this GI niche may better reflect the influence of intestinal microbes on the host and disease.

The *Lactococcus* genus was more abundant in the cecal contents of mice fed PDs than in mice fed GBDs. Reports have demonstrated that *Lactococcus* in rodent gut microbiotas results from the industrial production of casein, a main protein source in PDs but not in GBDs, and that the DNA is an artifact of casein production rather than detection of viable bacteria living in the intestinal tract (31). As high-throughput sequencing of the 16S rRNA gene is unable to distinguish between live and dead microbes, we are unable to determine the source of *Lactococcus* in our study but DNA fragments resulting from casein production are a potential explanation for the observed increased abundance in the ceca of mice consuming PDs relative to GBDs.

*Akkermansia* spp. are mucin-degrading bacteria that reside in the mucosal layer and exhibit anti-inflammatory properties. When broken down, mucin constitutes energy sources that can be used by the gut microbiota (32). Increased relative abundance of *Akkermansia* may reflect increased mucin production in the GI tract. As *Akkermansia* had a higher relative abundance in PDs than in GBDs, our study suggests that the PDs may promote an anti-inflammatory environment in the gut. In contrast, *Alistipes* were more abundant in mice consuming the LabDiet 5001 GBD. As this genus has been reported to be both beneficial and detrimental to host health (33), understanding the impact of GBDs on the gut microbiota requires further investigation.

Although many steps were taken to reduce the variably and increase rigor in this study, it is not without limitations. Specifically, only male mice were included in this study in order to reduce variability by using a homogenous experimental population. We acknowledge that this approach does not consider sex as a biological variable and as sex can influence microbiota-associated mouse phenotypes (34), we encourage our data to be replicated in female mice. Additionally, our study did not determine whether gut microbiota-derived metabolites are associated with the observed phenotypes. Indeed, as short-chain fatty acids (SCFA) are the primary metabolites produced from microbial fermentation of fiber and as these metabolites impact the mammalian host in rodent and human studies, we posit that SCFA availability may explain the observed fiber-associated morphometry phenotypes.

There are multiple potential mechanisms by which dietary fiber could influence intestinal morphometry. First, the fermentative product butyrate is a primary energy source for colonocytes and prevents autophagy in the colonic epithelium (35). Second, SCFA stimulate goblet cell mucus production to support a healthy gut morphology (36). Third, butyrate helps maintain the integrity of the intestinal barrier via promoting tight-junction protein expression (37–39). Further investigation of these molecular mechanisms to explain the fiber-induced changes in intestinal morphometry will be crucial for developing rodent diets that optimize GI health and foster diverse microbial communities.

### Conclusion

In conclusion, mice fed PDs with high soluble fiber content (≥ 75% of total fiber, ~7 gm% total diet) best recapitulated the GI morphometry of mice fed GBDs, but no PD recapitulated the gut microbial composition of mice fed GBDs. Our results confirm that diets are an important experimental variable and GBDs are an inappropriate control for diet-based studies. Despite the limitations of using GBDs in diet-based research, our study highlights that GBDs confer benefits to GI health and microbial richness relative to PDs, even with multiple fiber sources. Further work to formulate a PD with a mixture of soluble and insoluble fibers that recapitulates these attributes of GBDs is highly desirable to improve both the reproducibility and the broader impact of preclinical rodent studies.

## MATERIALS AND METHODS

### Animals

Six-week-old male C57BL/6J mice were purchased from Jackson Laboratory (Bar Harbor, ME; *n* = 30 mice). Mice were housed in ventilated cages (Tecniplast Green Line Caging: Tecniplast, Italy) on a 12 hour light/dark cycle (lights on at 7AM, off at 7PM), with *ad libitum* access to autoclaved water and irradiated food. All autoclaved cages contained bedding (ALPHA-driTM bedding: Shephard Specialty Papers, USA), a hut, and a nestlet. Temperature was maintained between 20°C and 25°C and humidity was monitored but not controlled. Mice were singly housed to avoid confounding cage effects and enable each mouse to be a biological replicate rather than a technical replicate for microbiota characterization (40). During the 7-day acclimation period, all mice were fed the GBD Teklad 2020X. Mice were then randomly assigned to one of the six diets for 28 days (*n* = 5 mice/group). Food consumption and body weight were measured on day 0 (when all mice were placed on Teklad 2020X), day 7 (when mice were randomly assigned to one of six diets), day 21, and day 35 (when the study terminated). Food was replaced and cages were changed on days 7 and 21. The study lasted a total of 35 days.

### Diets

All six diets used in this study contained sufficient essential vitamins and minerals and all had a similar macronutrient profile. Descriptions of the diets, including dietary fiber compositions, fiber ratio, and caloric content, are listed in Table 1.

**TABLE 1:** Composition of experimental diets

Product #	D11112225		D11112201		D11112231		D11112232	
Diet group name	*100C*		*75C/25I*		*25C/75I*		*25C/I/G/P*	
	**gm%**	***kcal%***	**gm%**	***kcal%***	**gm%**	***kcal%***	**gm%**	***kcal%***
Protein	18.8	*20*	19.0	*20*	19.3	*20*	19.3	*20*
Carbohydrate	61.1	*65*	61.6	*65*	62.7	*65*	62.7	*65*
Fat	6.5	*15*	6.5	*15*	6.7	*15*	6.7	*15*
Total		*100*		*100*		*100*		*100*
kcal/gm	3.78		3.81		3.88		3.88	
**Ingredient**	**gm**	***kcal***	**gm**	***kcal***	**gm**	***kcal***	**gm**	***kcal***
Casein	200	*800*	200	*800*	200	*800*	200	*800*
L-Cystine	3	*12*	3	*12*	3	*12*	3	*12*
Corn starch	390.5	*1,562*	381	*1,524*	362.3	*1,449*	362.3	*1,449*
Maltodextrin 10	110	*440*	110	*440*	110	*440*	110	*440*
Dextrose	150	*600*	150	*600*	150	*600*	150	*600*
Cellulose	100	*0*	75	*0*	25	*0*	25	*0*
Inulin^*a*^	0	*0*	25	*37.5*	75	*112.5*	25	*37.5*
Pectin^*b*^	0	*0*	0	*0*	0	*0*	25	*37.5*
Glucomannan^*b*^	0	*0*	0	*0*	0	*0*	25	*37.5*
Soybean oil	70	*630*	70	*630*	70	*630*	70	*630*
Mineral Mix S10026	10	*0*	10	*0*	10	*0*	10	*0*
Dicalcium phosphate	13	*0*	13	*0*	13	*0*	13	*0*
Calcium carbonate	5.5	*0*	5.5	*0*	5.5	*0*	5.5	*0*
Potassium citrate, 1 H2O	16.5	*0*	16.5	*0*	16.5	*0*	16.5	*0*
Vitamin mix V10001	10	*40*	10	*40*	10	*40*	10	*40*
Choline bitartrate	2	*0*	2	*0*	2	*0*	2	*0*
Yellow dye #5, FD&C^*c*^	0.025	*0*	0.025	*0*	0	*0*	0	*0*
Red dye #40, FD&C	0.025	*0*	0	*0*	0.025	*0*	0	*0*
Blue dye #1, FD&C	0	*0*	0.025	*0*	0.025	*0*	0.05	*0*
**Total**	**1,080.55**	***4,084***	**1,071.05**	***4,084***	**1,052.35**	***4,084***	**1,052.35**	***4,084***

^*a*^ Inulin assigned caloric value of 1.5 kcal/gm (per Roberfroid, J. Nutr. 129: 1436S–1437S, 1999).

^*b*^ Pectin and glucomannan assigned caloric value of 1.5 kcal/gm (predicted).

^*c*^ Food, Drug and Cosmetic.

The two GBDs were Teklad 2020X (Inotiv; Madison, WI) and LabDiet 5001 (Richmond, IN). LabDiet 5001 was chosen as it was the same grain-based diet used in the study by Chassaing et al. (19) and was found to have a profoundly different gut morphology compared with the PD containing cellulose, a PD similar to the 100C diet used in the current study. Teklad 2020X was selected to compare it with LabDiet 5001, as it is formulated by an independent company (Inotiv), has a distinct ingredient profile, and is phytoestrogen-free (i.e., excludes alfalfa and soybean). Teklad 2020X contains approximately 24 kcal% protein, 16 kcal% fat, and 60 kcal% carbohydrate, while LabDiet 5001 contains both alfalfa and soybean meal and approximately 29 kcal% protein, 14 kcal% fat, and 57 kcal% carbohydrate. Fiber contents of the GBDs were analyzed by Medallion Labs using the Association of Official Analytical Chemists method 991.43 (modified) to determine total, insoluble, and soluble fiber (LabDiet 5001: 21.1% total, 15.4% insoluble, 5.7% soluble; 2020X: 16.7% total, 12.3% insoluble, 4.4% soluble) (41).

The four PDs were gifted from Research Diets, Inc. (New Brunswick, NJ) and were based on the Open Standard Diet D11112201. These PDs contained varying compositions of insoluble fiber (cellulose) and soluble fibers (inulin, glucomannan, and pectin). The diets were 100C (100% cellulose), 75C/25I (75% cellulose, 25% inulin), 25C/75I (25% cellulose, 75% inulin), and 25C/I/G/P (25% cellulose, 75% mix of inulin, glucomannan, and pectin). To achieve isocaloric PDs, we added corn starch as needed to replace calories derived from soluble fibers.

Cellulose was chosen as a control fiber as it is non-fermentable and is commonly used in PDs, including the American Institute of Nutrition AIN-93 series diets. Inulin was selected as a soluble fiber source to be consistent with studies by Chassaing et al. and Griffin et al. (19, 22), where it was shown to increase cecal and colonic weight. The inulin used was derived from chicory root, with an average degree of polymerization of 23–25, a DP range of 2–60, and a purity of ≥98.5%. These specifications were based on chemical composition data from the manufacturers. The diet with multiple soluble fibers (inulin, pectin, and glucomannan) was designed to mimic the complexity of fibers expected in GBDs. Pectin was included as it is commonly found in GBDs (42), while glucomannan, a source of mannans, is typically found in both GBDs used in this study.

The fiber sources used in the PDs were carefully selected and analyzed. In PDs, the cellulose source is Solka Floc FCC 200—which provides 95.55% total fiber with 4.14% moisture and 0.31% ash (based on specification data from J. Rettenmaier USA LP). Orafti High Performance Inulin (Orafti North America, Inc.) contains ≥99.5% inulin in all batches (on a dry basis) with only 3% moisture and <0.2% ash—such that the total fiber content is 97%. Pectin 1400 (Tic Gums) provides 80% total fiber (all soluble) with 12% moisture and 8% ash. Glucomannan (Konjac Root, NOW Foods) has 97.5% fiber content. Medallion Labs (Minneapolis, MN, USA) tested all fibers used in this study and reported that cellulose provided 98.3% total fiber (96.5% insoluble), pectin provided 94.7% total fiber (93.3% soluble), inulin provided 96.2% total fiber (all soluble), and glucomannan provided 97.5% fiber (79.8% soluble and 17.7% insoluble).

### Fecal and tissue sample collection

Fresh fecal pellets were obtained from mice on days 0, 7, and 35 and stored at −20°C. On day 35, animals were anesthetized with isoflurane and then euthanized by cervical dislocation. To isolate gonadal fat, we made a midline incision to access the abdominal cavity. Each testicle was visualized, and adipose tissue directly attached was carefully excised to exclude noncontiguous fat depots. Small intestine and colon lengths were assessed using a tape measure. Cecum weight and gonadal fat mass were determined using a digital scale. Cecal and colon contents were collected, snap frozen in liquid nitrogen, and stored at −80°C. Small intestine length, colon length, cecum weight, and gonadal fat mass were measured.

### DNA extraction

Genomic DNA was isolated from fecal pellets, cecal contents, and colon contents using a phenol-chloroform extraction method followed by a DNA clean-up. One fecal pellet, two colon pellets, or 100 mg of cecal contents were suspended in 750 µL of lysis buffer (200 mM NaCl, 100 mM Tris, pH 8.0, 20 mM EDTA, and 20 mg/mL lysozyme) with 300 mg of 0.1 mm glass beads (BioSpec, Bartlesville, OK). Samples were vortexed briefly and incubated at 37°C for 30 min. For each sample, 85 µL of 10% SDS and 20 µL of proteinase K (15 mg/mL) were added and the mixture was incubated for another 30 min at 60°C. Bacterial cells were physically disrupted by adding 500 µL of 25:24:1 solution of phenol:chloroform:isoamyl alcohol and homogenizing in a bead beater (TeSeE Precess 48 Homogenizer, Bio-Rad, Hercules, CA) for 90 s at 5,300 rpm. Samples were then centrifuged at 13,000 rpm for 5 min at room temperature. The resulting supernatant was further purified with sequential washes with 500 µL of 25:24:1 phenol:chloroform:isoamyl alcohol and 500 µL of 100% chloroform. Finally, DNA was precipitated with 1,000 µL of 100% ethanol and 50 µL of 3 M sodium acetate at −80°C for 60 minutes and then cleaned using the Qiagen QIAamp DNA Stool Mini Kit (Qiagen, Valencia, CA) per manufacturer's instructions.

### 16S rRNA gene sequencing

Following DNA extraction, two consecutive polymerase chain reactions (PCR) were performed to amplify the V4 variable region of the 16S rRNA gene. The first PCR contained 120 ng of template DNA, six forward primers, six reverse primers (10 µM), and Robust DNA polymerase from the KAPA2G Robust PCR Kit (Kapa Biosystems, Wilmington, MA) (43). Cycling conditions were as follows: 95°C for 3 min; (95°C for 30 s; 50°C for 30 s; 72°C for 30 s) × 10 cycles; 72°C for 5 min. The second PCR further amplified the V4 variable region and added the Illumina MiSeq adapter primers and a single 12-nucleotide Golay error-correcting barcode for multiplexing (44). The KAPA HiFi HotStart ReadyMix reagent (Kapa Biosystems, Wilmington, MA) was used to amplify sequences from 5 µL PCR product generated by the first reaction. Cycling conditions were as follows: 95°C for 3 min; (95°C for 30 s; 50°C for 30 s; 72°C for 30 s) × 22 cycles; 72°C for 5 min. PCR products were purified using the HighPrep PCR clean-up kit (MagBio, Lausanne, Switzerland) and a DynaMag-96 side magnet (Life Technologies, Carlsbad, CA) per manufacturer's instructions. Final purified products were quantified and pooled at equimolar concentrations for high-throughput sequencing on the Illumina MiSeq platform (Illumina, San Diego, CA) using the MiSeq Reagent Kit v3 at the University of North Carolina at Chapel Hill high-throughput sequencing facility.

### 16S rRNA amplicon data processing

16S rRNA gene sequence read classification was performed using the Quantitative Insights into Microbial Ecology 2 pipeline (QIIME2, version 2022.2) (45). Reads were demultiplexed and primers and adapters were removed. Divisive Amplicon Denoising Algorithm (DADA2) was used to correct for Illumina-sequenced amplicon errors and to generate absolute sequence variants (SV) at a 100% identity threshold (46). Sequences were truncated at 217 base pairs. To eliminate rare taxa, we filtered out SV with lower than 0.01% of total reads for each run. Samples with fewer than 10,000 reads were removed to ensure that samples fell within a 10-fold sequencing depth range. Rarefication of data was carried out and a >10-fold variation in sequencing depth was observed across samples (47). To avoid loss of data from data rarefication for the 145 out of 147 samples with >10,000 sequences, we removed two samples with <10,000 sequences. Sequencing depth, number of samples sequenced, and total number of SV post filtering for each timepoint and sample site is detailed in Table S3.

Ultimately, 29 cecal samples, 29 colon samples, and 30 fecal samples were successfully characterized and analyzed. The SILVA classifier (release 138, 99% OTUs) performed taxonomic assignments (48). MicrobiomeAnalyst (49) calculated α-diversity using Shannon diversity and the absolute number of SV, β-diversity using Bray-Curtis dissimilarity indices, relative abundance of bacterial genera sampled from the three GI niches, and differential abundance of bacterial genera detected in the cecum of mice on different diets with LEfSe (50). Multidimensional scaling (MDS) plots were generated in R (version 4.3.0) using the vegan (version 2.6.4) package.

### Statistical analysis

Average daily calorie intake, body weight change, gonadal fat mass, small intestinal and colon length, and cecum weight were compared between experimental groups using a one-way ANOVA with Tukey's post-hoc test. PERMANOVA established statistical differences based on Bray-Curtis dissimilarity distances between diet groups and GI niches. A one-way ANOVA with Tukey's post-hoc test assessed differences in α-diversity measures between diet groups and GI niche. Primary taxonomic analysis was carried out at the genus level (reflecting the most refined taxonomic level for these data). The top genera identified by LEfSe most likely to explain differences between groups and significant pairwise comparisons were determined by DESeq2 (version 3.18) (51). An adjusted *P* value <0.05 was used as a threshold for significance for all statistical tests.

## Data Availability

Sample and raw sequence data are publicly available at the NCBI Short Read Archive (SRA) under BioProject ID PRJNA1159846.

## SUPPLEMENTAL MATERIAL

Figure S1 aem.01552-24-s0001.pdf MDS plot using Bray-Curtis dissimilarity distances for fecal samples collected at day 0 (A) and day 7 (B). Microbial communities are colored by diet group. No statistical significance was observed between any group (PERMANOVA).

Table S1 aem.01552-24-s0002.pdf LEfSe analysis of cecal microbial communities among diet groups.

Table S2 aem.01552-24-s0003.pdf FDRq values for pairwise comparisons between diets for the top six genera identified by LEfSe analysis.

Table S3 aem.01552-24-s0004.pdf Sequencing output based on site and timepoint.

ASM does not own the copyrights to Supplemental Material that may be linked to, or accessed through, an article. The authors have granted ASM a non-exclusive, world-wide license to publish the Supplemental Material files. Please contact the corresponding author directly for reuse.
