# Supplementary figures and images for: Purified diets containing high levels of soluble fiber and grain-based diets promote similar gastrointestinal morphometry yet distinct microbial communities
Source: Appl Environ Microbiol. 2024 Oct 24;90(11):e01552-24. doi: 10.1128/aem.01552-24 (PMC11577796; doi:10.1128/aem.01552-24)

SUPPLEMENTAL FIGURE 1

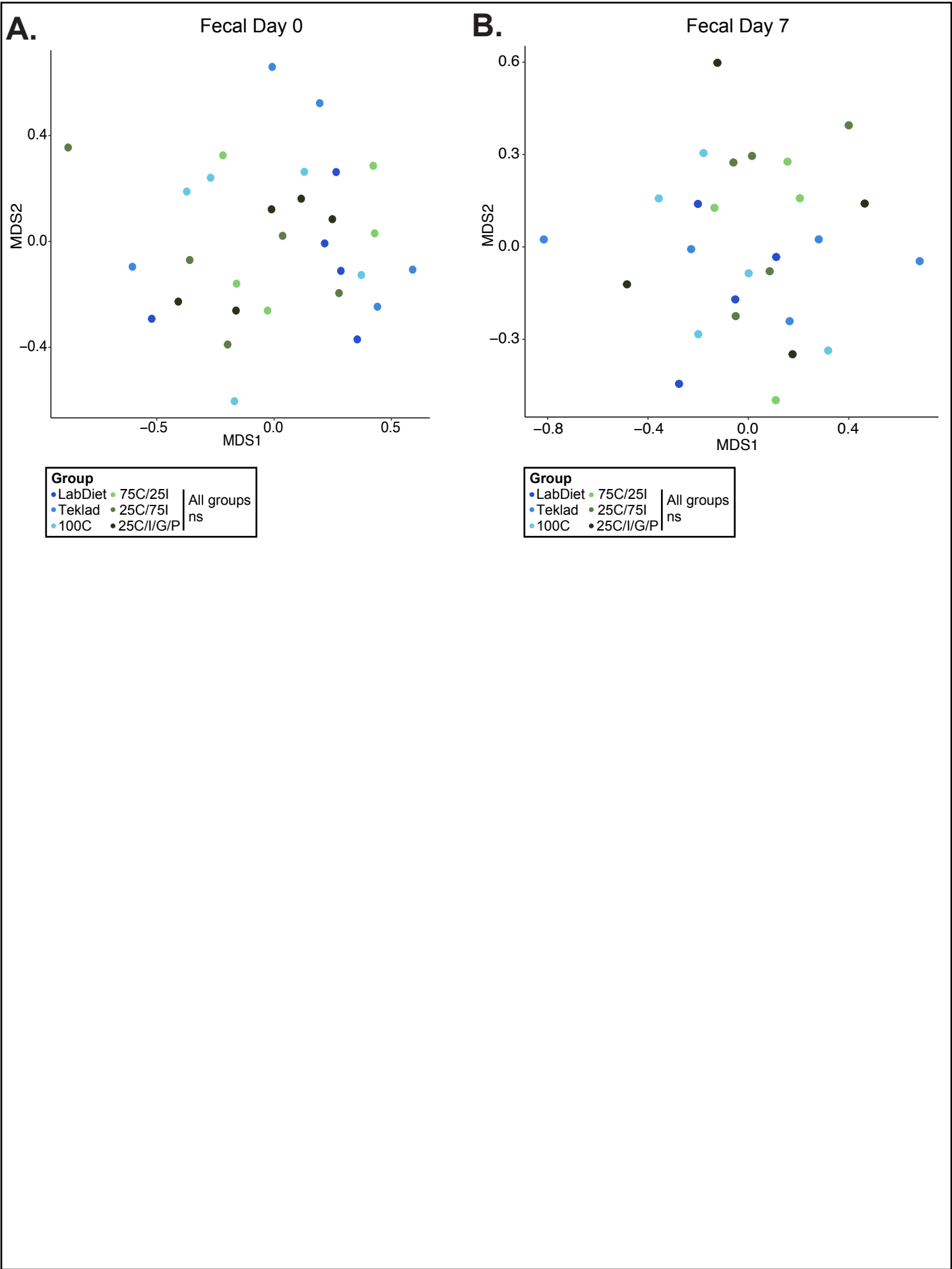

Supplement: Figure S1 — MDS plot using Bray-Curtis dissimilarity distances for fecal samples collected at day 0 (A) and day 7 (B). Microbial communities are colored by diet group. No statistical significance was observed between any group (PERMANOVA). [file aem.01552-24-s0001.pdf]
