# Supplementary material for: Purified diets containing high levels of soluble fiber and grain-based diets promote similar gastrointestinal morphometry yet distinct microbial communities
Source: Appl Environ Microbiol. 2024 Oct 24;90(11):e01552-24. doi: 10.1128/aem.01552-24 (PMC11577796; doi:10.1128/aem.01552-24)
Supplement: Table S1 — LEfSe analysis of cecal microbial communities among diet groups. [file aem.01552-24-s0002.pdf]

Genus	LDAscore	p value	FDRq value	Purina	Teklad	100C	75C/25I	25C/75I	25C/I/G/P
Family XIII UCG 001	4.06	0.000	0.003	13.45	14.50	11.08	10.38	0.00	8.39
Lactococcus	5.42	0.000	0.003	9.65	9.08	19.02	18.40	17.39	16.78
Tyzzerella	4.20	0.000	0.003	14.95	6.81	0.00	0.00	0.00	0.00
Not Assigned	5.83	0.000	0.004	20.49	20.06	18.68	18.50	17.50	16.98
Alistipes	5.76	0.000	0.004	20.18	18.05	15.66	16.71	14.55	17.01
Harryflintia	3.45	0.001	0.004	11.29	0.00	12.48	10.63	9.86	0.00
Akkermansia	5.72	0.001	0.004	15.37	17.30	19.35	18.59	20.06	19.74
UCG 005	3.67	0.001	0.004	13.18	11.97	0.00	0.00	0.00	6.66
Eubacterium xylanophilum group	5.46	0.001	0.004	17.52	19.15	13.47	13.05	9.39	9.68
Monoglobus	4.76	0.001	0.004	16.81	14.32	11.30	12.21	9.46	11.20
Anaeroplasma	4.27	0.001	0.006	15.20	11.19	11.64	8.66	0.00	6.07
Colidextribacter	4.95	0.001	0.006	17.01	17.08	17.60	16.44	15.54	14.46
Romboutsia	5.61	0.001	0.007	8.00	14.97	19.65	18.30	8.40	9.48
Incertae Sedis	5.01	0.002	0.007	17.30	17.52	17.46	17.76	14.21	15.50
Lachnoclostridium	5.37	0.002	0.007	19.24	18.60	18.76	18.23	17.31	17.25
uncultured	5.66	0.002	0.007	20.00	19.55	20.17	19.56	18.83	18.02
Lachnospiraceae NK4A136 group	5.95	0.002	0.007	19.89	20.97	18.27	20.76	20.85	17.92
Blautia	5.87	0.002	0.007	18.43	18.27	20.53	17.33	14.36	15.44
UCG 009	3.97	0.002	0.007	14.19	12.67	12.45	11.48	0.00	8.02
Intestinimonas	4.53	0.003	0.007	15.53	15.42	16.29	15.51	14.28	13.59
Clostridia vadinBB60 group	4.92	0.003	0.008	17.37	13.19	15.00	13.56	15.17	10.86
Parasutterella	4.86	0.003	0.008	14.53	11.93	17.19	14.13	16.07	15.14
Eubacterium ventriosum group	4.82	0.003	0.008	11.52	17.00	10.22	10.17	0.00	8.91
Eubacterium brachy group	3.69	0.003	0.008	13.08	13.44	12.42	12.45	10.38	11.42
Lachnospiraceae UCG 006	5.44	0.006	0.014	19.16	16.72	17.54	17.39	14.82	16.11
Erysipelatoclostridium	5.10	0.006	0.014	12.62	14.91	14.39	17.96	14.13	17.40
Ruminococcus	4.26	0.007	0.014	12.96	15.16	0.00	0.00	0.00	10.37
Oscillibacter	4.90	0.007	0.014	17.37	16.88	17.21	16.06	15.93	13.41
Lachnospiraceae UCG 004	3.95	0.007	0.014	9.04	13.11	14.15	13.08	7.82	9.40
Turicibacter	5.21	0.007	0.014	15.00	18.31	16.15	16.15	0.00	8.31

Genus	LDAscore	p value	FDRq value	Purina	Teklad	100C	75C/25I	25C/75I	25C/I/G/P
Enterorhabdus	4.85	0.009	0.016	17.54	17.46	15.55	15.79	16.20	16.17
Roseburia	5.30	0.009	0.016	16.93	17.47	18.67	17.60	16.26	13.64
Marvinbryantia	4.58	0.010	0.017	15.61	14.06	16.15	16.36	14.63	12.93
Erysipelotrichaceae	4.30	0.010	0.017	15.29	12.23	12.93	9.67	0.00	8.78
GCA 900066575	5.09	0.011	0.018	16.46	15.53	17.86	18.01	16.25	14.15
Tuzzerella	3.88	0.012	0.020	10.10	11.23	13.94	10.46	9.40	8.89
Bifidobacterium	6.02	0.013	0.020	13.90	18.12	11.08	16.09	18.54	20.98
Erysipelatoclostridiaceae	3.60	0.016	0.024	11.79	11.43	12.95	9.62	0.00	11.33
Ruminococcaceae	4.87	0.021	0.031	17.18	13.01	14.99	11.97	0.00	11.27
RF39	4.37	0.021	0.031	15.17	14.87	14.05	15.59	11.00	11.74
Bacteroides	5.35	0.032	0.045	18.10	17.04	19.14	18.36	17.64	17.77
Acetatifactor	5.14	0.035	0.049	16.14	16.11	18.18	15.78	14.92	14.24
Clostridium sensu stricto 1	3.94	0.050	0.068	0.00	0.00	11.61	14.10	0.00	0.00
Lachnospiraceae FCS020 group	3.90	0.058	0.077	14.49	13.84	14.08	14.44	14.31	12.78
A2	5.02	0.074	0.096	17.85	17.37	16.99	16.91	17.52	14.80
Muribaculaceae	5.69	0.084	0.104	19.72	19.37	18.95	20.50	20.41	19.40
Dubosiella	6.30	0.085	0.104	13.11	19.95	17.79	20.46	21.22	21.93
Anaerotruncus	4.79	0.090	0.109	16.82	14.35	17.03	15.73	16.29	13.30
NK4A214 group	3.12	0.112	0.133	10.97	11.50	11.90	10.65	10.21	10.98
Lactobacillus	5.51	0.124	0.144	15.45	16.91	13.25	16.69	19.33	18.38
Eubacterium coprostanoligenes group	4.61	0.190	0.216	15.03	6.81	16.31	13.55	11.76	14.55
Clostridia UCG 014	4.39	0.198	0.221	15.96	14.65	13.79	15.34	13.89	15.57
Parvibacter	3.02	0.274	0.300	10.07	9.42	10.78	0.00	0.00	11.02
UCG 010	3.65	0.440	0.473	12.59	13.40	12.63	11.82	10.92	12.81
Lachnospiraceae UCG 001	4.65	0.456	0.480	16.16	16.46	13.37	7.46	0.00	15.01
Eubacterium nodatum group	4.20	0.494	0.512	11.95	10.90	15.05	12.50	10.92	11.88
Defluviitaleaceae UCG 011	3.66	0.528	0.537	10.78	12.06	13.02	13.41	10.75	11.59
ASF356	3.46	0.863	0.863	12.50	11.75	11.04	12.65	10.62	9.50
