# Supplementary material for: Purified diets containing high levels of soluble fiber and grain-based diets promote similar gastrointestinal morphometry yet distinct microbial communities
Source: Appl Environ Microbiol. 2024 Oct 24;90(11):e01552-24. doi: 10.1128/aem.01552-24 (PMC11577796; doi:10.1128/aem.01552-24)
Supplement: Table S2 — FDRq values for pairwise comparisons between diets for the top six genera identified by LEfSe analysis. [file aem.01552-24-s0003.pdf]

Genera	LabDiet_vs_Teklad	LabDiet_vs_100C	LabDiet_vs_75C/25I	LabDiet_vs_25C/75I	LabDiet_vs_25C/I/G/P	Teklad_vs_100C	Teklad_vs_75C/25I	Teklad_vs_25C/75I	Teklad_vs_25C/I/G/P	100C_vs_75C/25I	100C_vs_25C/75I	100C_vs_25C/I/G/P	75C/25I_vs_25C/75I	75C/25I_vs_25C/I/G/P	25C/75I_vs_25C/I/G/P
Akkermansia	0.02	0.00	0.00	0.00	0.00	0.02	0.11	0.00	0.00	0.79	0.08	0.03	0.03	0.01	0.88
Alistipes	0.02	0.00	0.00	0.00	0.03	0.01	0.25	0.01	0.88	0.39	1.00	0.00	0.29	0.16	0.01
Family_XIII_UCG_001	0.21	0.00	0.00	0.00	0.00	0.00	0.00	0.00	0.00	0.91	0.00	0.22	0.00	0.36	0.05
Harryflintia	0.00	NA	0.63	0.65	0.00	0.00	0.00	0.00	1.00	0.19	0.09	0.00	0.99	0.01	0.01
Lactococcus	0.80	0.00	0.00	0.00	0.00	0.00	0.00	0.00	0.00	0.81	0.52	0.21	0.88	0.48	0.70
Tyzzerella	0.02	0.00	0.00	0.01	0.01	0.74	0.72	0.96	0.97	1.00	1.00	1.00	1.00	1.00	1.00
