# Supplementary material for: Purified diets containing high levels of soluble fiber and grain-based diets promote similar gastrointestinal morphometry yet distinct microbial communities
Source: Appl Environ Microbiol. 2024 Oct 24;90(11):e01552-24. doi: 10.1128/aem.01552-24 (PMC11577796; doi:10.1128/aem.01552-24)
Supplement: Table S3 — Sequencing output based on site and timepoint. [file aem.01552-24-s0004.pdf]

Sample site	Timepoint	1. Total samples sequenced	2. Median sequence depth	3. Total SVs	4. Total samples post filtering	5. Median sequence depth post filtering	6. Total SVs post filtering
Cecum	Day 35	30	73,092	480	29	50,303	289
Colon	Day 35	29	77,242	447	29	53,226	291
Feces	Day 0 + Day 7	58	68,994	1329	57	46,526	357
Feces	Day 35	30	48,409	580	30	29,610	358
Sites combined	Day 35	89	65,758	790	88	45,248	391
